# In Vitro Antibacterial Potential of *Salix babylonica* Extract against Bacteria that Affect *Oncorhynchus mykiss* and *Oreochromis* spp.

**DOI:** 10.3390/ani10081340

**Published:** 2020-08-03

**Authors:** Lenin Rangel-López, Adrian Zaragoza-Bastida, Benjamín Valladares-Carranza, Armando Peláez-Acero, Carolina G. Sosa-Gutiérrez, Helal F. Hetta, Gaber El-Saber Batiha, Ali Alqahtani, Nallely Rivero-Perez

**Affiliations:** 1 Instituto de Ciencias Agropecuarias, Área Académica de Medicina Veterinariay Zootecnia, Universidad Autónoma del Estado de Hidalgo, Rancho Universitario Av. Universidad km 1, EX-Hda de Aquetzalpa, Tulancingo, Hidalgo 43600, Mexico; ralolenin@gmail.com (L.R.-L.); pelaeza@uaeh.edu.mx (A.P.-A.); carolina_sosa@uaeh.edu.mx (C.G.S.-G.); 2 División Académica en Ciencias Agropecuarias, Universidad Juárez Autónoma de Tabasco, carretera Villahermosa-Teapa Kilómetro 25+2 Rancheria la Huasteca 2da sección, Villahermosa 86298, Mexico; 3 Facultad de Medicina Veterinaria y Zootecnia Universidad Autónoma del Estado de México, El Cerrillo Piedras Blancas, Toluca 50295, Mexico; benvac2004@yahoo.com.mx; 4 Department of Medical Microbiology and Immunology, Faculty of Medicine, Assiut University, Assiut 71515, Egypt; helal.hetta@uc.edu; 5 Department of Pharmacology and Therapeutics, Faculty of Veterinary Medicine, Damanhour University, Damanhour 22511, Egypt; gaberbatiha@gmail.com; 6 Department of Pharmacology, College of Pharmacy, King Khalid University, Guraiger, Abha 62529, Saudi Arabia; amsfr@kku.edu.sa

**Keywords:** *Salix babylonica* extract, in vitro, antibacterial activity, *Oncorhynchus mykiss*, *Oreochromis* spp.

## Abstract

**Simple Summary:**

Aquaculture development is limited by several diseases associated with bacteria that have developed resistance to antibiotics. In this context, new treatment alternatives are necessary. The aim of the present study was to evaluate the in vitro antibacterial effects of *Salix babylonica* hydro-alcoholic extract against some bacteria that affect rainbow trout (*Oncorhynchus mykiss*) and tilapia (*Oreochromis* spp.). The results indicate that *Salix babylonica* hydro-alcoholic extract has an antibacterial effect and could be an alternative treatment in diseases caused by microorganisms resistant to drugs in aquaculture.

**Abstract:**

Aquaculture development is limited by bacteria associated with several diseases; antibiotics are used for the treatment of these affections, but bacteria have developed resistance to these drugs. It is important to develop effective treatments that allow the production of antibiotic-free food. The aim of the present study is to evaluate the in vitro antibacterial effects of *Salix babylonica* hydro-alcoholic extract (SbHE) against *Aeromonas hydrophila*, *Listonella anguillarum*, *Edwarsiella tarda,* and *Streptococcus iniae*, bacteria that affect *Oncorhynchus mykiss* and *Oreochromis* spp. production. SbHE was obtained through the maceration technique. Reference strains were used and their sensitivity to antibiotics was determined. Minimal inhibitory concentration (MIC) and minimal bactericidal concentration (MBC) of SbHE were determined. Results showed that three of four evaluated bacteria were multidrug resistant, except *S. iniae*. SbHE showed antibacterial activity against all bacteria. Results indicate an MIC of 1.56 to 25 mg/mL and an MBC of 3.12 to 100 mg/mL. The greatest inhibitory activity occurred against *L. anguillarum* obtaining a MIC of 1.56 mg/mL and an MBC of 3.12 mg/mL. Results indicate that SbHE has bactericidal activity against *A. hydrophila*, *L.* *anguilalurm*, and *S. iniae* as well as bacteriostatic activity against *E. tarda* and could be an alternative treatment against these bacteria.

## 1. Introduction

Aquaculture is an agropecuary activity with growth potential primarily within the commercial alimentary industry, with a world production of 80 million tons annually [1]. In 2017, Mexico produced approximately 404 thousand tons of aquaculture products. Of these, the highest yielding species were tilapia and trout [2].

It has been observed that aquatic organisms are more susceptible to infectious agents, when density is increased, and water as well as food quality is decreased [3]. Infectious diseases, mainly those associated with bacteria, represent a great challenge in fish farming, since they affect fish growth, increase mortalities, and require treatment, which generate significant economic losses [4].

Vaccines, immunostimulants, chemotherapeutics, and antibiotics have been used for many years for the control and treatment of bacterial diseases. The use of chemotherapeutics and antibiotics are undesirable since they accumulate in the muscles, contaminate aquatic environments, stimulate the development of bacterial resistance, and increase production costs. As such, their use has been restricted in some countries [5,6,7,8]. Commonly used antibiotics in the treatment of bacterial infections in aquaculture are enrofloxacin, florfenicol, oxytetracycline, chlortetracycline, ciprofloxacin, norfloxacin, oxolinic acid, perfloxacin, sulfamethazine, gentamicin, and tiamulin [9,10].

Some bacteria that affect aquaculture is *Aeromonas hydrophila,* a Gram-negative bacterium, with a cosmopolitan distribution, that thrives in freshwater as well as marine environments. *A. hydrophila* causes skin ulcerations, hemorrhagic septicemia (red sore disease, red rot disease, and squamous protrusion disease). Multi-drug resistance of this bacterium to antibiotics has been previously reported [11,12,13].

*Listonella anguillarum* is a Gram-negative bacterium that affects rainbow trout (*Oncorhynchus mykiss*) which causes erythema around the fins and mouth, lethargy, appetite loss, skin color change, ulcers on the integument, injuries in abdominal muscles, liver, gills, spleen, and kidneys [14].

On the other hand, *Edwarsiella tarda*, a Gram-negative bacterium that affects tilapia (*Oreochromis* spp.) causes capillary hyperemia, exudation, focal petechial hemorrhages, leukocytosis, and hemorrhagic septicemia. Furthermore, it also produces lesions in the intestine and skin as well as exophthalmia [15]. Finally, *Streptococcus iniae* a Gram-positive bacterium that affects *Oreochromis* spp., causes meningoencephalitis, lethargy, back stiffness, erratic behavior in swimming, and death [16]. Antibiotic resistance to multi-drug treatments has been reported for both pathogens [17].

Multidrug resistance (antibacterials) in aquaculture systems requires that alternative therapies be explored for the treatment of diseases caused by these agents. Among these alternatives, the use of plant extracts has been proposed; due to their content of secondary compounds such as alkaloids, terpenes, tannins, saponins, glycosides, and flavonoids, they have shown antibacterial activity in studies in vitro and in vivo in fish and shellfish [18]. On the other hand, it has been demonstrated that plant extracts are safer, more environmentally friendly, inexpensive, accessible, and easier to prepare than commercial antibiotics. Most importantly, they present fewer side effects over treated organisms, and are overall safer [19,20].

The antibacterial activity of plant extracts has been demonstrated against some bacterial strains of significance in aquaculture production. Tkachenko et al. determined the in vitro antibacterial activity of ethanolic extract from leaves of *Ficus* spp. using the disk diffusion method, against *A. hydrophila*, isolated from *O. mykiss* [12]. Their results indicate that the extract presents an intermediate inhibitory activity (10–12 mm of inhibition halos) on the growth of this bacterium. Boran et al. evaluated the in vitro antibacterial activity of Green tea (*Camellia sinensis*) with the disk diffusion method against *L. anguillarum* [21]. They demonstrated the susceptibility of this bacterium to the extract, with an inhibition diameter of 18 mm.

Baba et al. infected tilapias with *E. tarda*, previously supplemented with *Citrus limon* essential oil at a concentration of 0.5% in commercial food [22], and survival rates of 63.33% after 20 days were observed. Gütepe et al. fed tilapias with *Thymus vulgaris*, *Rosmarinus officinalis,* and *Trigonella foenum* extracts throughout 45 days [23]. They were then infected with *S. iniae*, resulting in a survival rate of 88% in fish fed *T. vulgaris* extract.

*Salix babylonica*, commonly known as weeping willow, is a tree that belongs to *Salicaceae* family, their leaves contain flavonoids, terpenoids, lignans, and phenolic compounds [24]. This species has been evaluated by different authors to determine its antibacterial activity against some bacteria of importance in human and animal medicine. In a study conducted in 2015 by Popova and Kaleva, weeping willow leaves were collected during different seasons, to evaluate its antibacterial activity against *Escherichia coli*, *Salmonella enterica*, and *Staphylococcus aureus*. They observed the best results occurred with leaves collected in June. These induced an antibacterial activity less than 100 mg/mL [25].

In this regard, Ali and Aboud [26] evaluated the methanolic extract of *S. babylonica* and its hot and cold water infusions. They determined that *Enterobacter* spp., *Klebsiella* spp., *A. hydrophila*, and *Shigella dysenteriae*, showed resistance to treatments evaluated independently of the extraction method. The methanolic extract inhibited the growth of *Pseudomonas aeruginosa* (50 mg/mL), *E. coli* (100 mg/mL), and *S. aureus* (50 mg/mL). The hot water infusion inhibited growth of these bacteria at a concentration of 100 mg/mL. González-Alamilla et al. [27] evaluated the hydroalcoholic extract of *S. babylonica* against *E. coli*, *Listeria monocytogenes,* and *S. aureus* obtaining a minimal inhibitory concentration (MIC) of 100 mg/mL, 50 mg/mL, and 25 mg/mL, respectively. In 2020 González-Alamilla et al. evaluated the methanolic extract of *S. babylonica* against *E. coli*, *Salmonella typhi*, *Salmonella choleraesuis*, and *P. aeruginosa* and determined a MIC of 100 mg/mL; 25 mg/mL for *S. aureus* and *L. monocytogenes* and a MIC of 12.5 mg/mL over *Bacillus subtillis* [28]. 

In accordance with the aforementioned, the aim of the present study was to evaluate the antibacterial effect of *Salix babylonica* hydro-alcoholic extract (SbHE) against *A. hydrophila*, *L. anguilalurm*, *E. tarda,* and *S. iniae*, bacteria that affect rainbow trout (*Oncorhynchus mykiss*) and tilapia (*Oreochromis* spp.) production.

## 2. Materials and Methods

### 2.1. Plant Material

Weeping willow (*Salix babylonica*) leaves were collected in the municipality of Tulancingo de Bravo, located in Hidalgo State, Mexico (20°05′09″ N 98°21′48″ W). The Herbarium of UNAM (National Autonomous University of Mexico) was consulted and the plant was verified as *Salix babylonica* L. (IBUNAM: MEXU: 9744).

### 2.2. Hydroalcoholic Extract Obtention

SbHE was prepared following the methodology described by González-Alamilla et al. [27]. First, 1000 g of weeping willow leaves (*Salix babylonica*) were collected, washed, and dried in shade at room temperature. Then, 250 g of dried leaves obtained were macerated in 1000 mL of hydroalcoholic solution (30% methanol and 70% water) over 48 h at room temperature and in light absence. Subsequently, the mixture was filtered with filter paper (Whatman^®^ 42, Darmstadt, Germany), and finally the extract was concentrated under reduced pressure in a rotary evaporator (Büchi R-300, Flawil, Switzerland).

### 2.3. Bacterial Strains and Culture Conditions

The strains used were *Aeromonas hydrophila* CAIM347, *Listonella anguillarum* CAIM 763, *Streptococcus iniae* CAIM527, and *Edwardsiella tarda* CAIM 1875. These were obtained from the Collection of Microorganisms of Aquatic Importance (CAIM) of the Center for Research in Food and Development of Mazatlán, Sinaloa, Mexico (CIAD). Strains were reactivated in Trypticase Soy Agar (DIBICO^®^, Mexico City, Mexico) and incubated at 30 °C and 37 °C (only *S. iniae*) for 24 h. After incubation a colony was inoculated in nutritive broth (Becton Dickinson and Difco Company, Chicago, IL, USA) and incubated at 30 °C and 37 °C for 24 h.

### 2.4. Test of Antibiotic Sensitivity

Antibiotic sensitivity was determined using the disk diffusion method in Muller–Hinton agar (DIBICO^®^ Mexico City, Mexico). Following the methodology described by the Clinical and Laboratory Standards Institute (CLSI) [29], 100 µL previously adjusted to a 0.5 McFarland standard (Remel, R20421, Lenexa, KS, USA) of each bacterial strain were inoculated and evenly distributed on the plate. After 15 min, multidiscs (PT-35, Mexico City, Mexico) were placed on the plate and incubated at 30 °C and 37 °C (only *S. iniae*) for 24 h. After incubation completion time, the inhibition halos were measured and were compared with the measures established by the CLSI. The antibacterial agents used were cephalotin (30 µg), cefotaxime (30 µg), ciprofloxacin (5 µg), chloramphenicol (30 µg), nitrofurantoin (300 µg), ampicillin (10 µg), carbenicillin (100 µg), gentamicin (10 µg), netilmicin (30 µg), norfloxacin (10 µg), sulfamethoxazole/trimethoprim (25 µg), and amikacin (30 µg).

### 2.5. Test of Antibacterial Activity

For determined SbHE antibacterial activity, the minimal inhibitory concentration (MIC) and the minimal bactericidal concentration (MBC) were obtained, in accordance with the CLSI guidelines [29]. To determine the MIC, microdilution in a plate was used. The concentrations evaluated of SbHE were 1.56, 3.12, 6.25, 12.50, 25, 50, 100, and 200 mg/mL; as a positive control we employed Kanamycin (0.03–4.0 µg/mL, AppliChem 4k10421, Darmstadt, Germany) and for the negative control we used nutritive broth (Becton Dickinson and Difco Company, Chicago, IL, USA). Treatments were evaluated in triplicate.

In a 96-well plate, 100 µL of SbHE concentrations were added, along with 10 µL of a bacterial cell suspension previously adjusted to a 0.5 McFarland standard. The plates were incubated at 30 °C for *A. hydrophila*, *L. anguillarum,* and *E. tarda* and at 37 °C for *S. iniae*, for 24 h. Once the incubation period elapsed, 20 µL of a 0.04% (w/v) p-iodonitrotetrazolium (Sigma-Aldrich 18377, St. Louis, MO, USA) solution were added into each well and incubated for 30 min at 30 °C and 37 °C. The MIC was determined by the concentration at which the solution turned pink [30]. 

To determine the minimal bactericidal concentration (MBC) prior to addition of p-iodonitrotetrazolium, 5 µL from each well were inoculated in Müller–Hinton agar (DIBICO^®^ Mexico City, Mexico) and incubated at 30 °C and 37 °C (*S. iniae*) for 24 h. The MBC was determined by the concentration at which no visible growth of the bacteria occurred [31].

### 2.6. Statistical Analysis

Data were obtained from the disk diffusion method, and MIC and MBC were normalized and analyzed using a variance analysis (ANOVA) to determine significant statistical differences between treatments. The difference between means was compared with Tukey’s test (*p* < 0.05) using SAS version 9.0 (SAS Institute, NC, USA)

## 3. Results

### 3.1. Test of Antibiotic Sensitivity

Results of antibiotic sensitivity showed that three of four bacterial strains (*A. hydrophila*, *L. anguillarum* and *E. tarda*) were resistant to at least three antibiotics. *A. hydrophila* and *E. tarda* were resistant to cephalotin, ampicillin, and carbenicillin. *L. anguillarum* was resistant to cephalotin, ampicillin, and amikacin and *S. iniae* was only resistant to ciprofloxacin (Table 1).

### 3.2. Test of Antibacterial Activity

The results showed the extract has the capacity to inhibit the growth of the strains evaluated. Nevertheless, significant statistical differences (*p* = 0.0001) were observed among them. The SbHE presented more effective inhibitory activity against *L. anguillarum* (1.56 mg/mL), followed by *E. tarda* (3.12 mg/mL), and finally *A. hydrophila* and *S. iniae* (25 mg/mL) (Table 2).

On the other hand, results of minimal bactericidal concentration of SbHE indicated that this extract has bactericidal effects against all evaluated bacteria (*p* = 0.0001). Greater bactericidal activity was observed against *L. anguillarum* (3.12 mg/mL) followed by *E. tarda* and *S. iniae* (25 mg/mL); of these, *A. hydrophila* (100 mg/mL) was the least susceptible to SbHE (Table 2).

## 4. Discussion

For many years antibacterials have been used indiscriminately in aquaculture which has led to the emergence of bacteria resistant or multiresistant to these drugs, a problem that has stimulated the search for functional, innocuous alternative, with low environmental impact. Plants and their derivatives comply with these characteristics and have been shown to induce antibacterial activity during in vitro and in vivo studies; as such, potentially leading to the development of an alternative for use in fisheries units [32].

As with other livestock activities fish farming is also affected by the presence of infectious diseases, mainly those associated with bacteria. *A. hydrophila*, *L. anguillarum*, *E. tarda,* and *S. iniae* have been linked with mortality ranges of 50% to 100% in aquaculture production systems of *O. mykiss* and *Oreochromis* spp. Clinical signs of infected individuals may occur or be asymptomatic and lead to sudden death [33,34,35].

In the present study, an antibiotic sensitivity test was performed with reference strains of *A. hydrophila*, *L. anguillarum*, *E. tarda,* and *S. iniae*. Results indicated that *A. hydrophila* is resistant to at least two antibiotics groups (cephalosporin: cephalotin and penicillin: ampicillin and carbenicillin). According to López-Pueyo et al. [36], this strain can be classified as multidrug resistant, since it presents resistance to more than one group of antimicrobials. The bacteria is of clinical importance and has the ability to generate epidemic outbreaks where it is present.

Wamala et al. [37] determined the sensitivity to antibiotics of bacteria belonging to the same genus (*Aeromonas* spp.) and observed resistance to ampicillin, corroborating the results of the present study. Meanwhile, Negrete et al. [38] identified different bacteria isolated from kidneys of *Carassius auratus*, including A. *hydrophila*, which was classified as multidrug resistant (resistant to cephalotin, tetracycline, nefilmezine, ampicillin, carbenicillin, and kanamycin), further supporting results of the present investigation in which this bacteria was resistant to cephalothin, ampicillin, and carbenicillin.

On the other hand, *L. anguillarum* showed resistance to cephalotin, ampicillin, and amikacin. According to the criteria outlined by Lopez-Pueyo et al. [36], this bacterium can be classified as a multidrug resistant strain. In a similar study carried out by Vaseeharan and Ramasamy, who isolated *L. anguillarum* from *Penaeus monodon* [39], resistance to ampicillin, chlortetracycline, streptomycin, and penicillin was reported, further reinforcing the resistance to ampicillin reported in the present study.

*E. tarda* showed the same resistance pattern as *A. hydrophila* (resistant to cephalothin, ampicillin, and carbenicillin). Nikapitiya et al. [40] carried out tests for sensitivity to antibiotics with bacteria isolated from *Danio rerio*, including *E. tarda*. That research reported that this bacterium was resistant to beta-lactams (penicillin, amoxicillin), further supporting our results.

*S. iniae* was a strain sensitive to most of the antibiotics used in the present study. Nevertheless, it was the only bacterium resistant to ciprofloxacin, coinciding with the results of Mukwabi et al., who isolated different bacteria from aquaculture production systems in Kenya [17]. *S. iniae* was among the bacteria isolated in that study and showed resistance to ciprofloxacin.

The strains used for the antibacterial evaluation of SbHE showed different degrees of sensitivity to antibiotics, most of them being multi-resistant to antibacterial agents. The evaluation of SbHE antibacterial activity against bacteria that affect aquaculture production of *O. mykiss*, showed that the extract can inhibit the growth of *A. hydrophila* (MIC of 25 mg/mL). Wei et al. evaluated in seeds and flowers the methanolic extract of *Michelia champaca* and determined that the concentration at which growth of *A. hydrophila* was inhibited [41] was at 31.3 and 15.6 mg/mL, respectively. These concentrations are similar to the ones obtained in the present study.

In this sense, SbHE inhibited growth of *L. anguillarum* at a concentration of 1.56 mg/mL. Bulfon et al. [42] analyzed 15 ethanolic extracts against *L. anguillarum*, and reported that only *Lavandula officinalis* and *Origanum vulgare* inhibited the growth of this bacteria at a concentration of 2.1 mg/mL, a greater concentration than that reported in the present study which could be due to the type and concentration of secondary metabolites present in *S. babylonica* leaves and the kind of extract used in both studies.

Determination of MIC of SbHE on bacteria that affect the production of *Oreochromis* spp., allowed us to determine that the growth of *S. iniae* was inhibited at 25 mg/mL. Abutbul et al. [43] evaluated ethanol, methanol, ethyl acetate, and methanol/ethyl acetate extracts from *Rosmarinus officinalis* against *S. iniae*, using the disk diffusion technique. The best results obtained were those treated with the ethyl acetate extract that presented an inhibition halo of 37.5 mm/mg. Nevertheless, these results are not totally comparable since the technique used was different from that used in the present experiment.

Regarding *E. tarda*, growth was inhibited by SbHE at a concentration of 3.12 mg/mL. In 2009 Lee and Najiah obtained a MIC of 15.6 mg/mL with *Citrus microcarpa* extract using an American Type Culture Collection (ATCC) strain of *E. tarda* and 7.8–31.3 mg/mL over field strains [44]. The MIC of SbHE was lower even when comparing our MIC with that obtained against *E. tarda* field strains. 

The minimal bactericidal concentration of SbHE against bacteria that affects *O. mykiss* and *Oreochromis* spp. production showed that SbHE has bactericidal effects against *L. anguillarum* (3.12 mg/mL), *E. tarda* and *S. iniae* (25 mg/mL), and *A. hydrophila* (100 mg/mL). Roomiani et al. [45] evaluated methanolic extracts of *Rosmarinus officinalis*, *Anethum graveolens*, *Zataria multiflora,* and *Eucalyptus globulus* against *S. iniae*, obtaining an MBC of 15.6 to 500 µg/mL, lower concentrations than those obtained in the present study. Nevertheless, the plants and kind of extracts differed, therefore the kind of compounds present and the activity of each one changed [46]. Currently the number of studies in which the MBC of plant extracts is determined is lacking, which limits the discussion of our results.

Calculations of the MBC/MIC ratio indicated that SbHE has bacteriostatic activity against *E. tarda* and bactericidal activity against *A. hydrophila*, *L. anguillarum,* and *S. iniae*. According to Djihane et al. [47], when the MBC/MIC ratio is greater than four the effect is bacteriostatic and when it is less than or equal to four the effect is bactericidal. When analyzing the results of the experiment performed by Roomiani et al., it was observed that the extracts of *Rosmarinus officinalis*, *Anethum graveolens*, *Zataria multiflora,* and *Eucalyptus globulus* have a bactericidal effect against *S. iniae*, corresponding with our results.

Some studies have established that the antibacterial activity of plant extracts is due to their content of secondary metabolites. Gligoric et al. analyzed different *Salix* extracts [48], finding that *S. babylonica* has phenolic compounds (20.17 mg) and flavonoids (3.13 mg). González-Alamilla et al. analyzed *S. babylonica* methanolic extract, and found the extract contains thymol (0.5319 mg/mL) and carvacrol (0.4158 mg/mL) [28].

Furthermore, Rivero-Pérez et al. [49] determined the partial chemical composition of *S. babylonica* hydroalcoholic extract, finding phenolic compounds, coumarins, lactans, sterols, triterpenes, flavonols, flavonoids, saponins, and floratanins, as well as linalol, thymol, and carvacrol, which were identified using gas chromatography. González-Alamilla et al. reported that the organic fraction of *S. babylonica* hydroalcoholic extract [27] contains the secondary metabolites with the highest antibacterial activity and determined that two flavonoids (luteolin and luteolin glucoside) are responsible for this activity.

Additionally, it has been reported in in vivo studies in sheep, goats, and rabbits that SbHE does not modify hematological parameters associated with the overall health status of animals under study. Nor does it kill *Artemia salina* exposed to the extract, at the doses and frequencies reported, so it could be inferred that the extract is nontoxic [49,50].

## 5. Conclusions

The present study showed that SbHE has bactericidal activity in vitro, against *A. hydrophila*, *L. anguilalurm,* and *S. iniae* and bacteriostatic activity against *E. tarda*, bacteria of significance in rainbow trout and tilapia production, used in the food industry. The greatest results obtained were those against *L. anguillarum*. Thus, it may be an alternative treatment against these bacteria commonly found in fisheries. To further explore the use of this alternative, however, it is necessary to carry out in vivo studies.

## Figures and Tables

**Table 1:** Results of inhibition halos (mm) and antibiotic sensitivity of *A. hydrophila*, *L. anguillarum*, *E. tarda,* and *S. iniae*.

Antibiotic	*A. hydrophila*	*L. anguillarum*	*E. tarda*	*S. iniae*
Cephalotin	14 (R)	14 (R)	14 (R)	35 (S)
Cefotaxime	30 (S)	26 (S)	30 (S)	30 (S)
Ciprofloxacin	25 (S)	25 (S)	30 (S)	15 (R)
Cloramphenicol	25 (S)	20 (S)	20 (S)	20 (S)
Nitrofurantoin	25 (S)	25 (S)	25 (S)	20 (S)
Ampicillin	6 (R)	6 (R)	11 (R)	30 (S)
Carbenicillin	6 (R)	17 (S)	18 (R)	30 (S)
Gentamicin	20 (S)	20 (S)	20 (S)	20 (S)
Netilmicin	20 (S)	15 (S)	22 (S)	20 (S)
Norfloxacin	20 (S)	20 (S)	20 (S)	25 (S)
Sulfamethoxazole/Trimethoprim	22 (S)	20 (S)	18 (S)	20 (S)
Amikacin	20 (S)	6 (R)	20 (S)	20 (S)

S: sensitive, R: resistant.

**Table 2:** Minimal inhibitory concentration and minimal bactericidal concentration of hydroalcoholic extract from *Salix babylonica* against evaluated bacteria.

Bacteria	Minimal Inhibitory Concentration		Negative Control	Minimal Bactericidal Concentration		Negative Control
	SbHE (mg/mL)	Positive Control (µg/mL)		SbHE (mg/mL)	Positive Control (µg/mL)	
*A. hydrophila*	25 ^c^	2 ^b^	NA	100 ^c^	4 ^c^	NA
*L. anguillarum*	1.56 ^a^	0.5 ^a^	NA	3.12 ^a^	0.5 ^a^	NA
*E. tarda*	3.12 ^b^	0.5 ^a^	NA	25 ^b^	0.5 ^a^	NA
*S. iniae*	25 ^c^	0.5 ^a^	NA	25 ^b^	1 ^b^	NA
*p*-Value	0.0001			0.0001		

SbHE = *Salix babylonica* hydroalcoholic extract. NA = No activity. ^a,b,c^ Different literals in the columns indicate significant statistical differences (*p* ≤ 0.05).
